# Use of massage therapy by mid-aged and older Australian women

**DOI:** 10.1186/s12906-022-03626-w

**Published:** 2022-05-30

**Authors:** Suzy Ladanyi, Jon Adams, David Sibbritt

**Affiliations:** 1 School of Nursing, Faculty of Health, University of Technology Sydney, PO Box 123, Broadway, Ultimo, NSW 2007 Australia; 2 Australian Research Centre in Complementary and Integrative Medicine (ARCCIM), School of Public Health, Faculty of Health, University of Technology Sydney, Ultimo, NSW 2007 Australia; 3 Australian Centre for Public and Population Health Research (ACPPHR), School of Public Health, Faculty of Health, University of Technology Sydney, Ultimo, NSW 2007 Australia

**Keywords:** Massage therapy, Massage therapist, Complementary and alternative medicine, Women

## Abstract

**Background:**

Massage is a widely acceptable and popular form of complementary medicine (CM) among Australian women. While there is some research that reports on massage use in younger women, there is minimal research exploring massage use in the treatment of chronic illness in older women. This study provides an estimate of the prevalence of massage use, as well as identifying the characteristics significantly associated with consultation with a massage therapist, for mid-age and older Australian women.

**Methods:**

A cross-sectional sub-study was conducted on a sample of women drawn from the *45 and Up Study*; a large cohort study of adults aged 45 years and over*.* Data from 1795 women were included in the analyses and massage use was compared against measures of demographics, health status and health care utilisation.

**Results:**

A total of 174 (7.7%) women consulted with a massage therapist in the previous 12 months. Women were more likely to consult a massage therapist if they have tertiary level education (O.R. = 1.67; 95% C.I.: 1.04, 2.65; *p* = 0.031), private health insurance (O.R. = 6.37; 95% C.I.: 4.41, 9.19; *p* < 0.001) and/or osteoarthritis (O.R. = 1.72; 95% C.I.: 1.19, 2.48; *p* = 0.004). They were also more likely to consult a massage therapist if they have a poorer health-related quality of life (HRQoL) (O.R. = 1.14; 95% C.I.: 1.04, 1.27; *p* = 0.007).

**Conclusion:**

Older, tertiary-level educated Australian women with private health insurance were more likely to use massage therapy, as were women with osteoarthritis specifically. Women with lower HRQoL were found to be more likely to use massage therapy in the treatment of their chronic illness. This research provides insight into the determinants of massage use among ageing women and is useful for governments in consideration of accessibility to holistic healthcare when developing public policy for healthcare in Australia.

## Background

Over recent decades, the use of complementary medicine (CM) has steadily increased globally including in the United States (US), United Kingdom (UK), Canada and Australia [1–5]. It has been well established that CM use is more popular among women [6, 7].

Massage therapy is one of the most popular CM modalities in many countries, [3, 8, 9]. Moreover, Australian-focused studies have identified massage therapy as the most popular and widely used form of CM among women [10–12]. Such research has reported the prevalence of consultation with a massage therapist in a 12-month period as 42% amongst young (18-23yrs) women and 25% amongst mid-aged (45-50yrs) women [13]. While international researchers have examined the prevalence and determinants of CM in mid-aged and older adults [14–17], and the efficacy of massage use in older adults [18], these studies do not report on the prevalence and determinants of massage use in mid-aged and older women specifically. In Australia, while some studies have reported on prevalence and determinants of massage use specifically [9, 13] as well as massage and associated HRQoL [19], more research is needed to better understand the determinants of use among mid-aged and older Australian women.

Massage therapy has the potential to benefit mental health and general well-being as it has been reported to alleviate stress and fatigue as well as promote sleep through relaxation and improving blood circulation, blood pressure [20, 21] and support tissue recovery and aid in the prevention of injury after exercise [22]. Additional to the mental health and general well-being benefits of massage therapy, it has also been found to be effective in the treatment of musculoskeletal pain, arthritic and general back pain, neck pain and hip pain [18, 21].

Massage therapy is becoming increasingly recognised for its role as a stand-alone treatment alongside the conventional treatment of disease and medical conditions as it has been reported to be a safe and effective intervention in alleviating blood pressure in the treatment of patients with hypertension [23, 24]; as well as reducing fatigue and promoting sleep during patient recovery from cardiopulmonary bypass graft surgery [25]. It has also been reported that general practitioners (GPs) more commonly refer patients to massage therapists, partly due to their believing in the efficacy of massage therapy [26, 27]. Despite these encouraging findings, women are still more likely to use conventional treatment only, the majority of the time, and less likely to integrate the use of massage therapy for medical conditions [26, 28, 29].

Previous research focused on young and mid-aged Australian women who utilise massage therapy, shows they are more likely to be married, to have a tertiary education, manage well on their income, and have private health insurance, than those not using massage [13]. This same research shows younger women are less likely to use massage if they report depression, anxiety, respiratory conditions and diabetes, and also if they smoke [13]. While the findings in this study are consistent with other research findings [5, 9, 30] and provided much needed information on massage use among young and mid-aged women; the nature of the survey questions utilised, meant that it was only possible to determine that the women had used massage, the questions did not however, indicate any specific condition or reason for such use.

## Methods

The *45 & Up Study* [approved by the University of New South Wales Human Research Ethics Committee (HREC)] is the largest cohort study undertaken in Australia and the Southern Hemisphere; with the aim being to provide insight into the health-related behaviours and needs of mid-age to older Australians. Conducted by the Sax Institute, prospective participants were randomly sampled from the Department of Human Services (then, Medicare Australia) enrolment database, resulting in population coverage of one in ten [31]. A total of 267,153 participants were recruited for baseline and follow-up questionnaires [31] with the baseline questionnaire administered between January 2006 and December 2009.

### Sample

The findings reported here are from a sub-study of the *45 and Up Study* undertaken between April and October of 2017. A project-specific questionnaire, developed to collect information about the health service utilisation of a sample of mid-age and older women drawn from the 45 and Up Study database was used. Participants in this study referred to as ‘women’, were assigned the sex of female at birth and identified as women. A total of 1,925 randomly sampled women aged between 53 and 95 (mean = 69) years returned a completed questionnaire, providing information of their demographics, health status and healthcare utilisation. Ethics approval for this sub-study dataset was gained from the HREC at the University of Technology Sydney, Australia.

### Demographic characteristics

Participants were asked about demographic information including age, area of residence, marital status, highest level of education, ability to manage on available income, and whether or not they had private health insurance. Area of residence was assigned according to the Accessibility/Remoteness Index of Australia (ARIA +) [32, 33]. Women were also asked about their level of activity relating to exercise as well as their consumption of alcohol and cigarettes/tobacco.

### Health status

Participants were asked to self-rate their HRQoL using the EQ-5D-3L, an instrument comprising five health dimensions: mobility, self-care, activities, pain/discomfort, and anxiety/depression. The EQ-5D is a widely used and standardised index validated in Australian research [34, 35]. Health-related hardiness and body mass index (BMI) were also included in the questionnaire. Hardiness relates directly to perceived health and how this applies to health-related behaviours and in turn, how individuals utilise healthcare services [36–38]. Finally, participants were asked to indicate if they were diagnosed or treated by a doctor in the past 12 months for 12 common age-appropriate symptoms, including: depression; anxiety/nervous disorder; dementia/Alzheimer’s disease; asthma; diabetes; osteoarthritis; osteoporosis; Parkinson’s disease; heart disease (including heart attack or angina); stroke; hypertension; and cancer (excluding skin cancer).

### Health service utilisation

Participants were asked to indicate the frequency with which they consulted healthcare practitioners in the past 12 months. A list of 10 conventional practitioner types were provided: general practitioner (GP); medical specialist; hospital doctor; nurse; pharmacist/chemist; counsellor; psychologist; dietician; physiotherapist; and occupational therapist. Meanwhile, 10 CM practitioner types were provided: massage therapist; chiropractor; osteopath; acupuncturist; naturopath/herbalist; homeopath; meditation instructor; yoga instructor; nutritionist; and Chinese medicine practitioner. Participants were also asked about their use of CM products and practices such as aromatherapy oils, herbal medicine, vitamins and minerals, meditation (without instructor), and yoga (without instructor).

### Statistical analysis

Bivariate analysis was used to examine the association between massage use and all of the demographic data, health status and health care utilisation measures. Specifically, chi-square tests were used to examine the association between massage use and categorical measures. Student’s t-test were used to examine the association between massage use and continuous measures. Multiple logistic regression model building was used to identify statistically significant characteristics associated with massage therapist consultations. Specifically, all variables with a bivariate *p*-value < 0.25 were entered into a multiple logistic regression model, then a backward stepwise approach was employed, using the log-likelihood, to determine the most parsimonious model predicting massage use. All statistical analysis was conducted using STATA 16.1.

## Results

A total of 174 (7.7%) women consulted with a massage therapist in the previous 12 months. Table 1 presents the association between consultation with a massage therapist and demographic characteristics. Women who have tertiary level education (*p* < 0.001) and/or private health insurance (*p* < 0.001) were more likely to consult a massage therapist in comparison to women without a tertiary education and/or private health insurance.

**Table 1:** The association between demographic characteristics and consultation with a massage therapist

Demographic	Consulted with a massage therapist				
	**Yes**		**No**		***p*-value**
	**n**	**(%)**	**n**	**(%)**	
**Area of Residence**					
Major Cities	65	(45)	842	(48)	
Inner Regional	64	(44)	682	(39)	
Outer Regional / Remote	15	(10)	213	(12)	0.454
**Marital Status**					
Single	9	(6)	141	(8)	
Married Defacto	95	(64)	1072	(61)	
Widowed/Divorced/Separated	44	(30)	548	(31)	0.614
**Education**					
No Formal Education or School	35	(24)	739	(42)	
Trade / Apprentice / Diploma	57	(39)	514	(29)	
University / Higher Degree	56	(38)	505	(29)	< 0.001
**Manages on Income**					
Little or no Difficulty	90	(60)	1190	(67)	
Some Difficulty	37	(25)	390	(22)	
Struggles with Income	22	(15)	185	(10)	0.149
**Private Health Insurance**					
Yes	92	(62)	356	(20)	
No	57	(38)	1420	(80)	< 0.001
**Exercise**					
Inactive / Sedentary	36	(24)	540	(32)	
Moderately Active	28	(19)	268	(16)	
Highly Active	83	(56)	887	(52)	0.16
**Cigarette/Tobacco Use**					
Yes	7	(5)	95	(5)	
No	141	(95)	1634	(95)	0.694
**Alcohol**					
Yes	9	(6)	147	(8)	
No	140	(93)	1629	(92)	0.337
**Sleep**					
Optimal	76	(52)	940	(54)	
Not Optimal	71	(48)	800	(46)	0.588
	**Mean**	**(SD)**	**Mean**	**(SD)**	**p-value**
EQ5D_THERMO	67.6	(17.8)	71.3	(18.8)	0.0180
Health Hardiness	30.2	(5.6)	30.2	(6.3)	0.9946
BMI	29.3	(7.0)	28.9	(7.3)	0.519

The association between use of a massage therapist and health status and is shown in Table 2. This data indicates that women who experience depression, anxiety/nervous disorder, dementia/Alzheimer’s disease, asthma, diabetes, osteoarthritis, osteoporosis, Parkinson’s disease, heart disease (including heart attack/angina), stroke, hypertension, cancer (Excluding skin cancer) are less likely to consult with a massage therapist, compared to women who do not experience these conditions (all *p* > 0.001).

**Table 2:** The association between health conditions and consultation with a massage therapist

Condition	Consulted a Massage Therapist				
	**Yes**		**No**		***p*-value**
	**n**	**(%)**	**n**	**(%)**	
**Depression**					
Yes	41	(28)	378	(21)	
No	108	(72)	1398	(79)	0.077
**Anxiety/Nervous Disorder**					
Yes	37	(25)	327	(18)	
No	112	(75)	1449	(82)	0.055
**Dementia / Alzheimer's Disease**					
Yes	0	(0)	7	(1)	
No	149	(100)	1796	(99)	0.443
**Asthma**					
Yes	28	(19)	413	(23)	
No	121	(81)	1363	(77)	0.213
**Diabetes**					
Yes	25	(17)	426	(24)	
No	124	(83)	1350	(76)	0.046
**Osteoarthritis**					
Yes	71	(48)	662	(37)	
No	78	(52)	1114	(63)	0.012
**Osteoporosis**					
Yes	40	(27)	414	(23)	
No	109	(73)	1362	(77)	0.329
**Parkinsons Disease**					
Yes	2	(1)	14	(1)	
No	147	(99)	1762	(99)	0.474
**Heart Disease (Incl heart attack/angina)**					
Yes	16	(11)	204	(11)	
No	133	(89)	1572	(89)	0.783
**Stroke**					
Yes	1	(1)	24	(1)	
No	148	(99)	1752	(99)	0.480
**Hypertension**					
Yes	55	(37)	611	(34)	
No	94	(63)	1165	(66)	0.536
**Cancer (excluding skin cancer)**					
Yes	5	(3)	97	(5)	
No	144	(97)	1679	(95)	0.270
**Other**					
Yes	47	(32)	518	(29)	
No	102	(68)	1258	(71)	0.541

Table 3 presents the association between consultation with a massage therapist and health service utilisation within the previous 12 months. These results indicate that women were more likely to consult with a massage therapist if they consulted a GP, medical specialist, pharmacist/chemist, psychologist and/or physiotherapist, compared to women who did not consult these practitioners (all *p* < 0.001).

**Table 3:** The association between health service utilisation measures and consultation with a massage therapist

Practitioner Consulted	Consulted a Massage Therapist				
	**Yes**		**No**		***p*-value**
	**n**	**(%)**	**n**	**(%)**	
**GP**					
0 Visits	26	(18)	586	(33)	
1–2 Visits	65	(44)	694	(39)	
3–6 Visits	37	(25)	393	(22)	
7 or more visits	21	(14)	103	(6)	< 0.001
**Medical Specialist**					
0 Visits	95	(64)	1461	(82)	
1–2 Visits	39	(26)	216	(12)	
3–6 Visits	13	(9)	75	(4)	
7 or more visits	2	(1)	24	(1)	< 0.001
**Hospital Doctor**					
Yes	13	(9)	95	(5)	
No	136	(91)	1681	(95)	0.085
**Nurse**					
Yes	6	(4)	147	(8)	
No	143	(96)	1629	(92)	0.065
**Pharmacist/Chemist**					
Yes	42	(28)	252	(14)	
No	107	(72)	1524	(86)	< 0.001
**Counsellor**					
Yes	10	(7)	59	(3)	
No	139	(93)	1717	(97)	0.033
**Psychologist**					
Yes	16	(11)	75	(4)	
No	133	(89)	1701	(96)	< 0.001
**Dietician**					
Yes	18	(12)	107	(6)	
No	131	(88)	1669	(94)	0.004
**Physiotherapist**					
Yes	50	(34)	162	(9)	
No	99	(66)	1614	(91)	< 0.001
**Occupational therapist**					
Yes	7	(5)	38	(2)	
No	142	(95)	1738	(98)	0.047

The associations between consultation with a massage therapist and consultations with another CM practitioner, as well as use of CM products and practices, can be seen in Table 4. This data shows that women who consult with a massage therapist are more likely to consult with a chiropractor, osteopath, acupuncturist, naturopath/herbalist, yoga instructor, nutritionist and/or Chinese medicine practitioner (all p < 0.001), compared to women who do not consult these practitioners. Also shown in Table 4, women who consult a massage therapist were also more likely to use aromatherapy oils; herbal medicine; homeopathic remedies; meditation; yoga (without instructor) and multivitamin (all *p* < 0.001), compared to women who do not use these products and practices.

**Table 4:** The association between use of complementary medicine and consultation with a massage therapist

Practitioner Consulted	Consulted a Massage Therapist				
	**Yes**		**No**		
	**n**	**(%)**	**n**	**(%)**	***p*-value**
**Chiropractor**					
Yes	28	(19)	75	(4)	
No	121	(81)	1701	(96)	< 0.001
**Osteopath**					
Yes	18	(12)	31	(2)	
No	131	(88)	1745	(98)	< 0.001
**Acupuncture**					
Yes	30	(20)	44	(2)	
No	119	(80)	1732	(98)	< 0.001
**Naturopath/Herbalist**					
Yes	16	(11)	32	(2)	
No	133	(89)	1744	(98)	< 0.001
**Homeopath**					
Yes	1	(1)	12	(1)	
No	148	(99)	1764	(99)	0.995
**Meditation Instructor**					
Yes	5	(3)	18	(1)	
No	144	(97)	1758	(99)	0.011
**Yoga Instructor**					
Yes	24	(16)	42	(2)	
No	125	(84)	1734	(98)	< 0.001
**Nutritionist**					
Yes	8	(5)	20	(1)	
No	141	(95)	1756	(99)	< 0.001
**Chinese Medicine**					
Yes	6	(4)	10	(1)	
No	143	(96)	1766	(99)	< 0.001
**Products or practices used**					
**Aromatherapy Oils**					
Yes	30	(20)	66	(4)	
No	119	(80)	1710	(96)	< 0.001
**Herbal Medicine**					
Yes	31	(21)	67	(4)	
No	118	(79)	1709	(96)	< 0.001
**Homeopathic Remedies**					
Yes	11	(7)	16	(1)	
No	138	(93)	1760	(99)	< 0.001
**Meditation (without instructor)**					
Yes	35	(23)	158	(9)	
No	114	(76)	1618	(91)	< 0.001
**Yoga (without instructor)**					
Yes	17	(11)	77	(4)	
No	132	(89)	1699	(96)	< 0.001
**Multivitamin**					
Yes	63	(42)	316	(18)	
No	86	(58)	1460	(82)	< 0.001

Table 5 presents the output from the logistic regression model building undertaken to determine the most parsimonious model which identifies the most important statistically significant predictors of consultation with a massage therapist. In comparison to participants who had no formal school education or school only education, those who had a trade/apprenticeship or diploma education were 1.83 (95% CI: 1.16, 2.88) times more likely to consult with a massage therapist, while those who had attained a university degree were 1.67 (95% CI: 1.04, 2.65) times more likely to consult with a massage therapist. Participants who had private health insurance were 6.37 (95% CI: 4.41, 9.19) times more likely to consult with a massage therapist, compared to those who did not have private health insurance that specifically included massage therapy rebate. Those participants who had been diagnosed or treated for osteoarthritis in the previous 12 months were 1.72 (95% CI: 1.19, 2.48) times more likely to consult with a massage therapist, compared to those who had not been diagnosed or treated for osteoarthritis in the previous 12 months. In terms of quality of life, as measured by the EQ-5D instrument, for every 10-point decrease in the EQ-5D score (i.e. a decline in quality of life), participants were 1.14 (95% CI: 1.04, 1.27) times more likely to consult with a massage therapist.

**Table 5:** Logistic regression model identifying the characteristics significantly associated with consultation with a massage therapist

Characteristics		Odds Ratio*	95% C.I	***p***-value
**Education**	No formal/School only	1	-	
	Trade/Apprentice/Diploma	1.83	1.16, 2.88	0.01
	University/Higher degree	1.67	1.04, 2.65	0.031
**Private Health Insurance**	No	1	-	
**(inc. rebate for massage therapy)**	Yes	6.37	4.41, 9.19	< 0.001
**Osteoarthritis**	No	1	-	
	Yes	1.72	1.19, 2.48	0.004
**EQ-5D**	10-point decrease	1.14	1.04, 1.27	0.007

^*^Adjusted odds ratios

## Discussion

This study reports on findings from a representative sample of mid-aged and older Australian women, focusing on the association between massage therapist consultation and demographic characteristics, health status, health service utilisation and CM utilisation. Four key findings emerged from the data. Findings identified that those with higher levels of education were more likely to consult a massage therapist; those with private health insurance which included a rebate for massage therapy were more likely to consult a massage therapist; out of all the diagnosis/conditions that we considered, only osteoarthritis was a significant predictor of massage use; and finally, the poorer the quality of life in mid-aged and older women, the more likely they were to consult a massage therapist.

This study found that among a cohort of mid-aged and older Australian women, 7.7% consulted a massage therapist in the previous 12 months. This result is in contrast with other studies that reported higher rates of massage use in Australian women. A previous study reporting on prevalence of massage use in young and mid-aged women found that 42% of young Australian women and 25% of mid-aged women consulted a massage therapist [13]. Another study reporting on the use of CM among 1800 Australian women aged 56-61 years, 51% consulted a massage therapist within the previous 12 months [19]. The contrast in findings here may be due to previous studies reporting on various set age ranges up to 61 years, where findings reported from this study included women 45 years and older. Massage therapy use however, was highest among those who used other forms of CM. This is consistent with other research data that indicates that users of one form of CM are likely to use multiple forms of CM [6].

This study also reported that women with a higher level of education are more likely to consult a massage therapist. These findings are consistent with reports from other Australian studies reporting on women’s use of massage therapy [39–44], as well as studies from the US [1, 45, 46], Canada [47], UK [48] and Sweden [49]. As reported here, women with private health insurance were more likely to consult a massage therapist, with this finding being supported by other research conducted in Australia [10, 42, 50]. The potential implication of this is that women who have better access to health care through private health insurance, including more choice and financial incentives through rebates, find it easier to access the services of massage therapy in the treatment of their chronic illness. Therefore, measures to improve accessibility for CM services such as massage in ageing populations could result in more holistic approaches to heath care in the future.

Osteoarthritis is a chronic disease of the joints that is degenerative with age. Conventional treatment options for people with osteoarthritis are limited to medications which are often high in toxicity and/or surgical intervention [51]. Often, despite treatment with conventional methods, those with osteoarthritis still live with debilitating pain and functional limitations that affect quality of life and activities of daily living. It is therefore not surprising that older adults with functional limitations [52], including those with musculoskeletal conditions and back/joint pain [13, 53, 54], are frequent users of massage therapy. Further, with the aging population, some of these consults may be due to underlying osteoarthritic pain.

Findings in this study indicate that osteoarthritis is a significant predictor of massage use in women 45 years and older; and women with osteoarthritis specifically, or who experience a decrease in their HRQoL more generally, were more likely to use massage therapy in the treatment of their chronic illness. Further, preliminary studies both qualitative and quantitative indicate massage therapy has been shown to be an effective and beneficial short-term therapy for people with osteoarthritis [51, 55, 56].

Although the efficacy of massage therapy in the treatment of osteoarthritis has not been proven definitively, there is some evidence to suggest that 60 min of massage may improve pain and functionality in these individuals [51]. Recipients reported feeling empowered and relaxed with improved quality of life when receiving massage for osteoarthritis [55]. Given there is limited research investigating the use of massage therapy in the treatment and care of people with osteoarthritis, this research indicating osteoarthritis is a significant predictor of massage use, together with reported positive outcomes in the benefits and use of massage therapy in the treatment and care of people with osteoarthritis [51, 56] provides reasonable grounds for further investigation of these correlations.

The findings reported here indicate that women increase their use of massage therapy as their quality of life decreases. This finding is consistent with other studies reporting that women with significantly poorer physical and emotional heath and poorer quality of life were more likely to seek massage therapy for their back pain and joint pain [19, 54]. A qualitative study reported participants experienced an improved quality of life associated with receiving massage therapy [55]. These findings are consistent with other research that incorporated quality of life measures while investigating women’s use of CM over time; women experiencing more illness were found to be more likely to use massage therapy and other forms of CM, than those experiencing no change or better health [50]. Finally, and perhaps in the promotion of a more holistic approach to overall well-being and quality of life during chronic disease and periods of poor quality of life, women may be more open to the idea that the benefits of massage therapy extend beyond the visceral. Massage therapy in many cases may be providing more than physical relief through mental health benefits during chronic illness [57–59].

While this research draws upon a robust and internationally recognised study sample, findings from this study should be interpreted with caution. The cross-sectional study design limits the ability to determine causality between variables. Also, participants in the 45 and Up Study cohort are by definition, restricted to a range specified by age. Therefore, when interpreting results, one must be mindful that the sample is representative of women aged 45 years and older and should not be generalised to women from younger age groups, or studies including both men and women, or only men. Another important point to consider is the possibility of selection bias, as people who agree to give up their time to complete surveys may view things differently to the remaining population. Finally, the questionnaires were conducted via self-report, and this has potential to introduce recall bias.

## Conclusion

The findings from this study suggest a considerable proportion of mid-aged and older women in Australia are consulting with a massage therapist and it is poorer quality of life and/or osteoarthritis that appears in part to be driving such massage therapy use. Women with a higher education who are able to access massage therapy via private health insurance, are more likely to consult with a massage therapist. Health care providers may use these findings in consideration of holistic care plans and well-rounded support for aging women. Finally, future research should investigate and consolidate evidence regarding massage therapy in pursuit of improved health outcomes related to massage use for mid aged and older women in our society.

## Data Availability

The data set could potentially be made available to other researchers if they obtain the necessary approvals. Further information on this process can be obtained from the 45 and Up Study (45andUp.research@ saxinstitute.org.au).

## Abbreviations

CM: Complementary medicine
HRQoL: Health-related quality of life
US: United States
UK: United Kingdom
HREC: Human Research Ethics Committee
ARIA +: Accessibility/Remoteness Index of Australia
EQ-5D: EuroQol Group—5 Dimension
BMI: Body mass index
GP: General practitioner
